# Brain Tumor Care in Relation to Patient Age—An Observational Study Between Years 2016 and 2022 in a Nationwide Cohort in Germany

**DOI:** 10.3390/curroncol33020104

**Published:** 2026-02-05

**Authors:** Frederic Bold, Gerardo Rico Gonzalez, Rüdiger Gerlach, Oliver Heese, Steffen K. Rosahl, Michael Stoffel, Juraj Kukolja, Frederick Palm, Emilia Machado Musri, Ali Allam, Ralf Kuhlen, Julius Dengler, Sven Hohenstein, Andreas Bollmann, Nora F. Dengler

**Affiliations:** 1 Faculty of Health Sciences Brandenburg, Medical School Theodor Fontane, Campus Bad Saarow, 15526 Bad Saarow, Germany; frederic.bold@helios-gesundheit.de (F.B.); gerardo.ricogonzalez@helios-gesundheit.de (G.R.G.); maria.machadomusri@helios-gesundheit.de (E.M.M.);; 2 Department of Anesthesiology, Intensive Care & Perioperative Pain Management, Helios Hospital Bad Saarow, 15526 Bad Saarow, Germany; 3 Department of Neurosurgery, Helios Hospital Bad Saarow, Pieskower Strasse 33, 15526 Bad Saarow, Germany; 4 Department of Neurosurgery, Helios Hospital Erfurt, 99089 Erfurt, Germany; ruediger.gerlach@helios-gesundheit.de (R.G.); steffen.rosahl@helios-gesundheit.de (S.K.R.); 5 Department of Neurosurgery, Helios Hospital Schwerin, 19055 Schwerin, Germany; 6 Department of Neurosurgery, Helios Hospital Krefeld, 47805 Krefeld, Germany; 7 Department of Neurology, Helios University Hospital Wuppertal, 42283 Wuppertal, Germany; 8 Faculty of Health, Witten/Herdecke University, 58455 Witten, Germany; 9 Department of Neurology, Helios Hospital Schleswig, 24837 Schleswig, Germany; 10 Helios Health Institute, 13125 Berlin, Germany; 11 Clinical Trial Management & Real World Data, Helios Health Institute, 13125 Berlin, Germany; sven.hohenstein@helios-health-institute.com (S.H.);

**Keywords:** brain tumor management, frailty, age, elderly

## Abstract

As societies continue to age, brain tumors increasingly affect older patients. Still, large-scale evidence on whether the relationship between age and brain tumor has been evolving over time is scarce. We examined longitudinal trends among different age groups of patients with brain tumors at 78 German hospitals. Two time periods were compared as follows: phase 1 (1 January 2016–31 December 2019; pre-pandemic) and phase 2 (1 January 2020–31 December 2022; pandemic). Patients were categorized as non-elderly (<65 years) or elderly (≥65 years), and according to 10-year age brackets. The clinical condition was quantified using the Elixhauser Comorbidity Index (ECI) and the Hospital Frailty Risk Score (HFRS). Among the 20,005 patients included, changes in characteristics of non-elderly/elderly patients over time behaved similarly, with improvements in ECI (19.3 to 18.4/15.2 to 14.3; each *p* < 0.01) and HFRS (2.1 to 1.6/4.7 to 4.1; each *p* < 0.01), and increases in rates of brain tumor resection (26.1% to 31.8%/22.7% to 27.8%; each *p* < 0.01). Only patients aged 75–84 years did not follow any of those trends. Over the examined 7-year period, general trends in brain tumor care in elderly subjects resembled those observed in non-elderly patients, except for those aged 75–84 years.

## Simple Summary

As societies continue to age, brain tumors increasingly affect older patients. Still, large-scale real-world evidence on the longitudinal evolution of the relationship between age and neuro-oncology is scarce. We used data from a nationwide hospital network in Germany to examine trends in brain tumor care for non-elderly vs. elderly patients between years 2016 and 2022. Among the 20,005 subjects included, trends in brain tumor care behaved quite similarly between non-elderly and elderly patients, given that, over time, both groups showed improvements in comorbidity and frailty profiles as well as an increase in rates of brain tumor resection. Only the specific cluster of those aged 75–84 years did not follow any of those trends. Our findings suggest similar trends in brain tumor care between non-elderly and elderly patients. They offer a rare glimpse into real-world dynamics of current neuro-oncological patient cohorts.

## 1. Introduction

The incidence of brain tumors is highest among patients older than 65 years [1]. In addition, in brain tumor care, as in clinical routine in general, the impact of frailty, an age-dependent syndrome of low resilience to external stressors, on outcomes has been gaining relevance, as societies continue to age [2,3,4]. The recent COVID-19-pandemic seems to have accelerated this process, as it led to a distinct increase in frailty in the general population due to reduced activity, mostly as a consequence of repeated lockdown measures [5,6].

Large-scale evidence on longitudinal trends in neuro-oncology in relation to age is scarce. The only recent observational study form Germany on this issue was published by our group [7]. We found that brain tumor patients admitted to hospitals between years 2016 and 2022 showed a decrease in frailty and comorbidities. However, in that study, there was no separate analysis according to age groups and types of brain tumors. We therefore designed a more granular subgroup analysis using nationwide data from Germany from the same period, aiming to delineate changes in patient and management characteristics according to different age groups and types of brain tumors.

## 2. Methods

### 2.1. Study Setting

Administrative data from all 78 hospitals within the Helios hospital network in Germany involved in the diagnosis and treatment of brain tumors were analyzed. The Helios hospital network serves rural and urban communities throughout all parts of Germany, caring for 7% of all in-hospital cases nationwide [8,9]. Patients with brain tumors were included in the study and grouped based on the time of hospital admission. The control group was derived from the phase of the pre-pandemic years from 1 January 2016 to 31 December 2019, referred to as phase 1. The study group consisted of patients from the pandemic years from 1 January 2020 to 31 December 2022, referred to as phase 2. The study was approved by the Ethics Committee of the University of Leipzig on 7 February 2022 (490/20-ek). Individual informed consent was waived, since this study is observational and presents no identity of enclosed patients.

### 2.2. Data Collection

To identify and group patients with brain tumors, the previously described classification introduced by the AANS/CNS Quality Outcomes Database Tumor Registry was used [10], based on the following International Classification of Diseases, 10th Revision (ICD-10) groups: C79.3 (intracranial metastases); C70.0, D32.0 (primary meningeal tumors); C71.0–C71.9 (primary high-grade/malignant brain tumors); D33.0–D33.2 (primary low-grade/benign brain tumors); and C75.1; D35.2; D44.3 (pituitary tumors). For the separate analysis of tumor types in those aged 65 years and older, we grouped these diagnoses according to the following categories: benign (primary meningeal tumors, primary low-grade/benign brain tumors, and pituitary tumors), malignant (primary high-grade malignant brain tumors), and metastases (intracranial metastases). The following operating procedures (OPS) codes categories served as classifiers for the types of management: craniotomy (5-010 and 5-012); brain tumor resection (5-015, 5-016, and 5-017); transfer to intensive care unit (ICU, 8-980, 8-98d, and 8-98f); and initiation of mechanical ventilation (8-70x and 8-71x). Age groups were defined as follows: non-elderly (<65 years) and elderly (≥65 years), and the following age brackets by years: <44, 45–54, 55–64, 65–74, 75–84, and >85. Among elderly patients, a calculation of the HFRS was performed based on ICD-10-Codes, as previously described [11]. According to the HFRS, patients older than 64 years can be categorized into previously established frailty groups: low (HFRS < 5), intermediate (HFRS 5–15), and high (HFRS > 15).

### 2.3. Statistical Analysis

Administrative data were extracted using QlikView (QlikTech, Radnor, PA, USA). Inferential analyses employed generalized linear mixed models (GLMMs) with hospitals as the random factor [12]. To estimate effects, the lme4 package (v1.1–21) in R (v4.0.2, 64-bit) was used [13,14]. All models included random intercepts, and significance was set at a two-sided 5% level. Cohort characteristics were compared using χ^2^ tests for categorical and ANOVA for continuous variables, reporting proportions, means, standard deviations (SD), and *p*-values. Logistic GLMMs with logit link assessed differences in treatment and outcome proportions, providing odds ratios (OR), confidence intervals (CI), and *p*-values. Daily case counts and frailty scores were analyzed using negative binomial models, with frailty scores multiplied by ten for integer scaling. The Elixhauser comorbidity index (ECI) and its items were calculated as previously reported [15]. For the weighted ECI, we applied the AHRQ algorithm. We performed the analysis of the outcome variable length of stay via the LMM based on a log-transformed dependent variable. Interaction analyses tested whether pre- to pandemic changes differed across frailty groups (reference: high frailty), using dummy-coded contrasts (low vs. high, and intermediate vs. high) and period coding of 0.5 (pandemic) vs. −0.5 (pre-pandemic).

## 3. Results

### 3.1. Non-Elderly vs. Elderly Subjects

Of the 20,005 included subjects hospitalized for brain tumors, 43.8% (*n* = 8759) were elderly. Patient and management characteristics for non-elderly and elderly patients are displayed in Table 1. Between phases 1 and 2, the proportion of elderly patients increased from 42.6% to 45.5% (*p* < 0.01). This was driven by a significant decrease in total numbers of hospitalizations for non-elderly subjects during phase 2, from 4.7 ± 3.3 to 4.0 ± 2.9 (*p* < 0.01), while total admissions of elderly subjects remained unchanged (3.5 ± 2.5 vs. 3.3 ± 2.4, *p* = 0.10).

The mean ECI among patients admitted for brain tumor care decreased between phases 1 and 2 both among the elderly (from 15.2 [SD 11.7] to 14.3 [SD 11.4], *p* < 0.01) and the non-elderly population (from 19.3 [SD 13.0] to 18.4 [SD 12.3] *p* < 0,01).

Similarly, the LOS decreased significantly both among elderly (from 11.4 days to 10.7 days, *p* < 0.01) and non-elderly patients (from 9.2 days to 8.5 days, *p* < 0.01). At the same time, the rates of brain tumor resection increased significantly among both age categories (elderly: 22.7% vs. 27.8%; non-elderly: 26.1% vs. 31.8%, each with *p* < 0.01). The same was observed for rates of transfer to ICU, which increased from 34.6% to 38.2% (*p* < 0.01) among the non-elderly and from 35.5% to 38.0% (*p* = 0.02) among the elderly. In-hospital mortality rates decreased significantly among non-elderly only, from 4.7% to 3.8% (*p* = 0.02), but not among the elderly (9.5% vs. 9.0%; *p* = 0.57).

### 3.2. Patient Characteristics per Age Brackets

Between phases 1 and 2, daily admissions decreased significantly for all age brackets, except patients aged 65–74 and >84, for which admissions remained unchanged (Table 2).

The decrease in daily admissions was most pronounced among age brackets 45–54 years (−23.1%) and <45 years (−15.4%), followed by 75–84 years (−14.3%). The mean ECI decreased for all age brackets, except those 75 years and older, which remained at baseline. The largest extent of this decrease was identified among the youngest and the oldest age brackets, with around −9% in those aged 54 and younger and about −8% in those >84 years.

**Figure 1:**
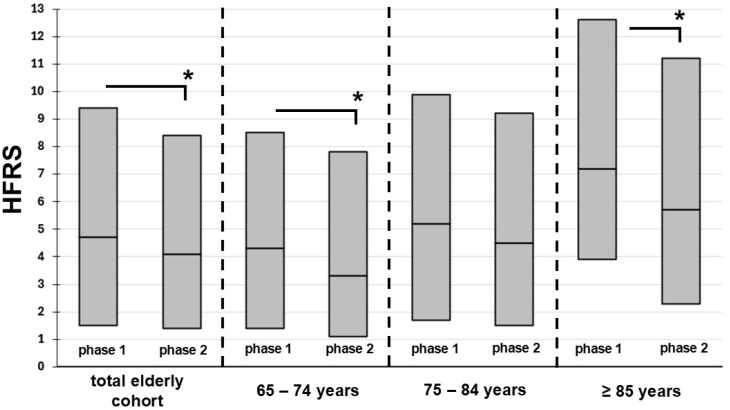
Frailty trends in all elderly brain tumor patients and according to elderly age brackets. HFRS, hospital frailty risk score. Boxes represent medians and IQR. The asterisk represents *p* < 0.05.

Figure 1 displays changes in HFRS between phases 1 and 2 for the elderly. In the total cohort of elderly patients, the median HFRS decreased from 2.1 to 1.6, (*p* < 0.01). HFRS remained unaltered only in those aged 75–84 years, while there was a significant decrease in all other age brackets. Among all elderly age brackets, the 75–84 years bracket was the only one without decreasing frailty levels between phases 1 and 2, while a quite substantial decrease in HFRS by more than 20% was observed in the other two elderly age brackets.

### 3.3. Management Characteristics per Age Brackets

Between phases 1 and 2, LOS remained unchanged among all age brackets except patients aged 55–74 years, for which a decrease in LOS was identified ranging between 8.0 and 11.6% (Table 3).

During phase 2, the rates of brain tumor resection increased for all age brackets quite substantially by a range of ORs between 1.3 and 2.9, except in those aged 75–84 years, for which no alterations were observed. Changes in rates of transfer to ICU were observed in patients aged <45 years, increasing by an OR of 1.3 (95% CI 1.1–1.5), and in those between 55 and 74 years, for which a significant decrease was observed by an OR of 1.2 (95% CI 1.1–1.4). In-hospital mortality rates remained unchanged, except in those aged 45–54 years, which displayed a significant decrease (OR 0.6 [95% CI 0.6–0.6]).

#### Management of Elderly Patients According to Tumor Entities

Among elderly patients, none of the three groups of tumor types displayed changes in daily admissions or in-hospital mortality rates between both phases (Table 4).

**Figure 2:**
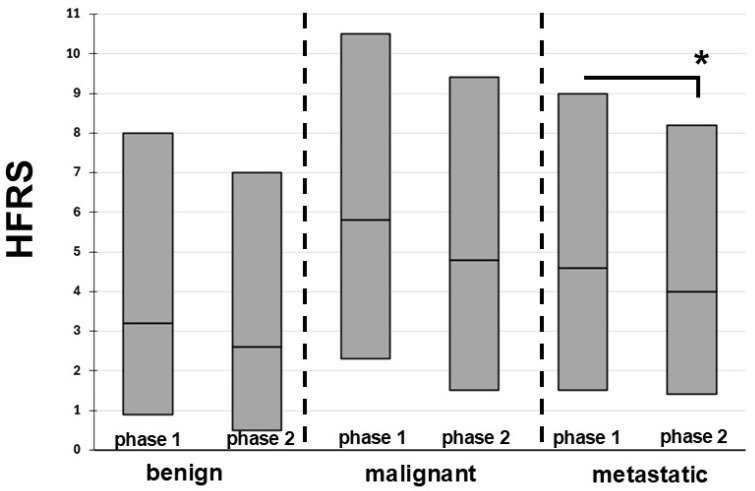
Frailty trends per tumor entity groups. HFRS, hospital frailty risk score. Boxes represent medians and IQR. The asterisk represents *p* < 0.05.

For all other variables, the most substantial changes were observed among patients with brain metastases. Here, significant decreases in the burden of comorbidities, frailty levels, and LOS were accompanied by an increase in the rate of tumor resection from 17.6% to 24.1%. Far less frequent alterations between phases 1 and 2 were identified in patients with malignant or benign brain tumors. For both of these entities, only one variable showed alterations, namely increased rates of tumor resection in benign and a decreased burden of comorbidities in malignant brain tumors. As displayed in Figure 2, changes in HFRS were observed only in the metastatic brain tumor cohort, with improvement from 4.6 (IQR 1.5–9.0) to 4.0 (IQR 1.4–8.2; *p* < 0.01).

## 4. Discussion

This study identified improvements in the burden of comorbidities, shorter LOS, and higher rates of surgery both among elderly and non-elderly brain tumor patients hospitalized in Germany during years 2020–2022, compared to years 2016–2019. However, in a more granular analysis of consecutive 10-year age brackets, certain age-dependent variations became apparent. Most prominently, among elderly patients, the specific group of subjects aged 75–84 years was the only group showing no improvement in levels of comorbidity or frailty, while all other elderly patients displayed a rather substantial drop in frailty by at least 20%. Second, the same age group was the only to display no change in rates of brain tumor resection, while this variable increased among all other age groups, even in non-elderly subjects.

### 4.1. Daily Admissions

As in most other medical fields, during the COVID-19-pandemic years 2020–2022, there was a significant decrease in absolute numbers of admissions of brain tumor patients, both for elderly and non-elderly subjects [7]. Daily admissions, however, decreased only in the non-elderly and those aged 75–84 years. Such selective processes at the thresholds of hospitals during the pandemic years in other medical disciplines are generally explained by certain groups being more hesitant to seek in-hospital care and potentially consulting with less specialized health facilities instead of hospitals in times of crisis [16]. Therefore, admission trends observed in our study may be explained by younger brain tumor patients having been more aware of or worried about potential in-hospital transmission of COVID-19 and having actively opted against hospitalization during the pandemic. Similarly, for very old patients, physicians outside of hospitals may have been less inclined to make referrals to shield this assumed high-risk group from potential contact with COVID-19 in hospitals. In the context of neurosurgery, our findings are in line with a study from Sweden, which showed that among elderly patients with neurosurgically treated disorders, the total numbers of neurosurgical procedures decreased significantly during the pandemic [17]. In addition, data from other European countries confirm a substantial decrease in neurosurgical case load during the pandemic, both in non-elderly and elderly subjects [18,19].

Interestingly, among elderly patients, daily admission trends did not differ across different brain tumor types, as there were no changes in daily admissions between phases 1 and 2 in any of the examined tumor entity groups. This is somewhat counterintuitive, given that one could have assumed that, in times of a pandemic, more elective benign cases would have been admitted to hospitals at lower rates than more urgent malignant or metastatic cases. The fact that such a differential triage was not observed in Germany may be explained by the country’s rather ample hospital bed capacity and the fact that there were barely any hospital shutdowns during the pandemic.

### 4.2. Comorbidities and Frailty

Previous evidence had already shown that, in Germany, patients admitted for brain tumors during the pandemic had a lower burden of comorbidities and lower frailty levels than before the pandemic [7]. Our study adds to this by describing this to be the case among non-elderly as well as elderly subjects. The fact that this trend was broken only by subjects aged 75–84 years, for which no improvement in comorbidities and frailty was observed, is of interest. In other words, this specific age group did not seem to be selected for healthier individuals at the thresholds of hospitals during the pandemic. In this context, it is of note that this age bracket is associated with the highest incidence rate of malignant brain tumors of all age groups [1]. Given that malignant brain tumors frequently necessitate urgent hospitalization, independent of whether a pandemic is ongoing, patients of this age group may have been less likely to defer clinical treatment than those of other ages.

Among elderly patients, the benign brain tumor group was the only to show no changes in the burden of comorbidities and frailty levels. In contrast, in phase 2, both the metastatic and the malignant brain tumor cohorts improved in the burden of comorbidities, and the metastatic group even in frailty levels. One could argue that these findings suggest that patients with benign brain tumors may have been less hesitant to present to hospitals during the pandemic. In contrast, more severe cases of brain tumors may have, due to less favorable comorbidity profiles and increased frailty, deferred hospitalization, resulting in a less comorbid and less frail hospital population among those more severe brain tumor types.

### 4.3. Rates of Brain Tumor Resection

Both among elderly and non-elderly subjects, there was an increase in rates of brain tumor resection. However, again, some age brackets stood out. By far the highest relative increase in rates of brain tumor surgery was present in those older than 84 years, for which an OR of 2.9 was identified, compared to an OR of 1.5 among those aged 65–74. A plausible reason could be the fact that, in the oldest age bracket, frailty levels improved significantly (from HFRS 7.2 to 5.7), which may have qualified more individuals within this group for surgery in phase 2, compared to phase 1.

Of note, among elderly patients, rates of tumor resection increased in benign as well as metastatic cases, while no significant change in rates was observed in malignant brain tumors. Given that daily admissions remained unaltered across all three different tumor type groups, this pattern of increase is noteworthy. A potential reason for constant rates of resection in malignant brain tumors, which mainly consist of high-grade astrocytoma and glioma, may be more objective criteria for surgery, compared to benign tumors and metastases, which may allow for more subjective leeway in deciding for or against surgery. In other words, during the pandemic, surgeons may have opted for surgery in those cases at higher rates. Another explanation may be that, during the pandemic, the burden of neurological deficits may have been higher in benign and metastatic cases, thereby necessitating surgical resection at higher rates.

### 4.4. LOS and Mortality Rates

Our study is the first study to show that LOS decreased in brain tumor patients during the pandemic phase both in elderly and non-elderly subjects. Interestingly, on closer inspection, this effect was exclusively present among the two age brackets from 55 to 74 years, for both of which the decrease in LOS ranged around 10%. Older and younger subjects showed no changes in LOS. This suggests that, during the pandemic, neurosurgeons may not have aimed for earlier discharges in young and very old subjects as much as in those aged 55–74 years. Of note, the decrease in LOS among elderly patients was driven mainly by earlier discharge of metastatic tumor cases, while the LOS among benign and malignant brain tumor cases remained unaltered. Overall, the observed trends in LOS may potentially represent a greater effort to reduce the risk of in-hospital transmission of COVID-19 for those aged 55–74 years with metastatic brain tumors.

Interestingly, in general, the overall shorter LOS was accompanied by no deterioration in mortality rates, which remained unchanged in the elderly, ranging between 9.0% and 9.5% and even improved in the non-elderly population (from 4.7% to 3.8%) and among elderly patients with metastatic brain tumors (from 12.3% to 9.7%). The fact that mortality rates among the elderly did not increase, even among the very old subgroup of those aged 85 and older, is noteworthy, given that their rates of surgery increased substantially, even doubled among the very old. This lack in increased mortality rates is somewhat contrary to recent evidence from Japan, collected through pre-pandemic years 2010 to 2015, which found that even in asymptomatic patients undergoing meningioma surgery increased age is highly associated with postoperative complications and mortality [20]. The comparably better results in our cohort may, again, be explained by the fact that the degree of improvement in frailty levels among the elderly in our study during the pandemic may have compensated for or even prevented an increase in mortality rates.

### 4.5. Limitations

Certain limitations exist within this study. First, data collection was retrospective in nature and, in general, analysis of administrative data is limited due to potential differences and errors in coding. Second, no information on tumor gradings, functional status, and long-term outcomes was available for analysis. Third, potential unmeasured confounders may include regional differences in treatment protocols and local case-volumes. Fourth, our analysis does not allow for identification of causal relationships between the examined variables. Fifth, our findings represent a nationwide cohort from a Western European country and may therefore not be generalizable to other regions.

## 5. Conclusions

The fact that both elderly and non-elderly brain tumor patients hospitalized in Germany during years 2020–2022 showed, with only few exceptions, improved levels of comorbidities and frailty, compared to years 2016–2019, suggests substantial similarities in trends across all ages. The observed improvements in clinical condition may have been a major driving force behind the simultaneous increase in rates of brain tumor surgery.

## Figures and Tables

**Table 1:** Patient and management characteristics in non-elderly and elderly brain tumor patients.

	Non-Elderly (*n* = 11,246)			Elderly (*n* = 8759)		
	Phase 1	Phase 2	*p*	Phase 1	Phase 2	*p*
Total number	6898	4348	<0.01	5128	3631	<0.01
Proportion per phase (%)	57.4	54.5	<0.01	42.6	45.5	<0.01
Daily admissions, mean (SD)	4.7 (3.3)	4.0 (2.9)	<0.01	3.5 (2.5)	3.3 (2.4)	0.10
ECI, mean (SD)	19.3 (13.0)	18.4 (12.3)	<0.01	15.2 (11.7)	14.3 (11.4)	<0.01
LOS, mean (SD)	9.2 (12.1)	8.5 (10.5)	<0.01	11.4 (11.3)	10.7 (10.7)	<0.01
Tumor resection, n (%)	1800 (26.1)	1383 (31.8)	<0.01	1164 (22.7)	1008 (27.8)	<0.01
In-hospital mortality, n (%)	302 (4.7)	150 (3.8)	0.02	428 (9.5)	281 (9.0)	0.57

**Table 2:** Patient characteristics in brain tumor patients per age brackets.

Age Groups, Years	Total Number	Daily Admissions, Mean (SD)	ECI, Mean (SD)
**<45**			
phase 1	1839	1.3 (1.3)	11.5 (9.5)
phase 2	1148	1.1 (1.2)	10.5 (9.4)
% change	−37.6	−15.4	−8.7
*p*-value	-	<0.01	<0.01
**45–54**			
phase 1	1908	1.3 (1.3)	15.0 (11.5)
phase 2	1059	1.0 (1.1)	13.6 (11.5)
% change	−44.5	−23.1	−9.3
*p*-value	-	<0.01	<0.01
**55–64**			
phase 1	3151	2.2 (1.8)	17.5 (12.4)
phase 2	2141	2.0 (1.7)	16.7 (11.8)
% change	−32.1	−9.1	−4.6
*p*-value	-	<0.01	<0.01
**65–74**			
phase 1	2782	1.9 (1.7)	19.1 (12.8)
phase 2	2043	1.9 (1.6)	17.9 (12.2)
% change	−26.6	0	−6.3
*p*-value	-	0.72	<0.01
**75–84**			
phase 1	2027	1.4 (1.4)	19.3 (13.2)
phase 2	1324	1.2 (1.2)	18.9 (12.7)
% change	−34.7	−14.3	−2.1
*p*-value	-	<0.01	0.38
**>84**			
phase 1	319	0.2 (0.5)	21.4 (13.0)
phase 2	264	0.2 (0.5)	19.7 (11.4)
% change	−17.2	0	−7.9
*p*-value	-	0.20	0.09

**Table 3:** Management characteristics in brain tumor patients per age brackets.

Age Groups, Years	LOS, Mean (SD)	Rate of Tumor Resection (% [n])	Rate of in-Hospital Mortality (%)
**<45**			
phase 1	7.4 (14.4)	24.3 (446/1839)	2.1
phase 2	6.9 (10.5)	28.9 (332/1148)	1.8
% change	−6.8	-	-
OR (95% CI)	-	1.3 (1.1–1.5)	0.8 (0.5–1.5)
*p*-value	0.06	<0.01	0.56
**45–54**			
phase 1	9.0 (10.7)	26.8 (511/1908)	4.7
phase 2	9.1 (11.0)	32.9 (348/1059)	3.2
% change	+1.1	-	-
OR (95% CI)	-	1.4 (1.2–1.7)	0.6 (0.6–0.6)
*p*-value	0.97	<0.01	<0.01
**55–64**			
phase 1	10.4 (11.2)	26.8 (843/3151)	6.4
phase 2	9.2 (10.2)	32.8 (703/2141)	5.2
% change	−11.6	-	-
OR (95% CI)	-	1.4 (1.2–1.6)	0.8 (0.6–1.0)
*p*-value	<0.01	<0.01	0.09
**65–74**			
phase 1	11.2 (11.2)	24.7 (686/2782)	8.2
phase 2	10.3 (10.8)	31.1 (636/2043)	7.0
% change	−8.0	-	-
OR (95% CI)	-	1.5 (1.3–1.7)	0.9 (0.7–1.1)
*p*-value	<0.01	<0.01	0.23
**75–84**			
phase 1	11.8 (11.6)	22.2 (450/2027)	10.8
phase 2	11.4 (10.9)	24.8 (329/1324)	11.2
% change	−3.4	-	-
OR (95% CI)	-	1.1 (1.0–1.4)	1.1 (0.8–1.4)
*p*-value	0.38	0.18	0.55
**>84**			
phase 1	10.9 (10.4)	8.8 (28/319)	13.2
phase 2	9.9 (9.0)	16.3 (43/264)	13.2
% change	−9.2	-	-
OR (95% CI)	-	2.9 (1.6–5.2)	1.1 (0.6–1.8)
*p*-value	0.12	<0.01	0.85

**Table 4:** Management of elderly brain tumor patients per tumor entity.

		Phase 1	Phase 2	*p*
Benign (*n* = 1933)	Total numbers	1134	799	-
	Daily admissions, mean	0.8	0.7	0.31
	ECI, mean (SD)	7.0 (9.5)	6.5 (9.7)	0.28
	LOS, mean (SD)	10.5 (12.5)	9.6 (10.9)	0.19
	Rate of tumor resection, % (*n*)	33.7 (382)	40.7 (325)	<0.001
	Rate of in-hospital mortality, % (*n*)	2.1 (22)	3.3 (24)	0.117
Malignant (*n* = 3199)	Total numbers	1862	1337	-
	Daily admissions, mean	1.3	1.2	0.4
	ECI, mean (SD)	15.4 (9.2)	14.7 (8.7)	0.03
	LOS, mean (SD)	11.9 (11.9)	12.0 (12.0)	0.78
	Rate of tumor resection, % (*n*)	21.8 (406)	24.2 (323)	0.07
	Rate of in-hospital mortality, % (*n*)	11.9 (177)	11.7 (135)	0.32
Metastatic (*n* = 3.627)	Total numbers	2132	1495	-
	Daily admissions, mean	1.5	1.4	0.13
	ECI, mean (SD)	29.2 (9.5)	28.0 (8.5)	<0.01
	LOS, mean (SD)	11.5 (10.0)	10.1 (9.2)	<0.01
	Rate of tumor resection, % (*n*)	17.6 (376)	24.1 (360)	<0.001
	Rate of in-hospital mortality, % (*n*)	12.3 (229)	9.7 (122)	0.024

## Data Availability

All original contributions presented in this study are included in the article. Further inquiries can be directed to the corresponding author.

## References

[1] Miller K.D., Ostrom Q.T., Kruchko C., Patil N., Tihan T., Cioffi G., Fuchs H.E., Waite K.A., Jemal A., Siegel R.L. (2021). Brain and other central nervous system tumor statistics, 2021. Cancer J. Clin..

[2] Alare K., Muili A., Afolabi S., Adetunji B., Aderinto N., Abdulla E. (2024). The Prognostic Utility of Frailty on the Outcomes of Primary Brain Tumor Surgery Patients: A Meta-Analysis. World Neurosurg..

[3] Bonney P.A., Chartrain A.G., Briggs R.G., Jarvis C.A., Ding L., Mack W.J., Zada G., Attenello F.A. (2021). Frailty Is Associated with In-Hospital Morbidity and Nonroutine Disposition in Brain Tumor Patients Undergoing Craniotomy. World Neurosurg..

[4] Torres-Perez P., Álvarez-Satta M., Arrazola M., Egaña L., Moreno-Valladares M., Villanua J., Ruiz I., Sampron N., Matheu A. (2021). Frailty is associated with mortality in brain tumor patients. Am. J. Cancer Res..

[5] Garner I.W., Varey S., Navarro-Pardo E., Marr C., Holland C.A. (2022). An observational cohort study of longitudinal impacts on frailty and well-being of COVID-19 lockdowns in older adults in England and Spain. Health Soc. Care Community.

[6] Yamada M., Kimura Y., Ishiyama D., Otobe Y., Suzuki M., Koyama S., Kikuchi T., Kusumi H., Arai H. (2021). The Influence of the COVID-19 Pandemic on Physical Activity and New Incidence of Frailty among Initially Non-Frail Older Adults in Japan: A Follow-Up Online Survey. J. Nutr. Health Aging.

[7] Hong B., Allam A., Heese O., Gerlach R., Gheewala H., Rosahl S.K., Stoffel M., Ryang Y.M., Burger R., Carl B. (2023). Trends in frailty in brain tumor care during the COVID-19 pandemic in a nationwide hospital network in Germany. Eur. Geriatr. Med..

[8] Nachtigall I., Lenga P., Jóźwiak K., Thürmann P., Meier-Hellmann A., Kuhlen R., Brederlau J., Bauer T., Tebbenjohanns J., Schwegmann K. (2020). Clinical course and factors associated with outcomes among 1904 patients hospitalized with COVID-19 in Germany: An observational study. Clin. Microbiol. Infect..

[9] Dengler J., Prass K., Palm F., Hohenstein S., Pellisier V., Stoffel M., Hong B., Meier-Hellmann A., Kuhlen R., Bollmann A. (2022). Changes in nationwide in-hospital stroke care during the first four waves of COVID-19 in Germany. Eur. Stroke J..

[10] Asher A.L., Khalafallah A.M., Mukherjee D., Alvi M.A., Yolcu Y.U., Khan I., Pennings J.S., Davidson C.A., Archer K.R., Moshel Y.A. (2021). Launching the Quality Outcomes Database Tumor Registry: Rationale, development, and pilot data. J. Neurosurg..

[11] Gilbert T., Neuburger J., Kraindler J., Keeble E., Smith P., Ariti C., Arora S., Street A., Parker S., Roberts H.C. (2018). Development and validation of a Hospital Frailty Risk Score focusing on older people in acute care settings using electronic hospital records: An observational study. Lancet.

[12] Baayen R.H., Davidson D.J., Bates D.M. (2008). Mixed-effects modeling with crossed random effects for subjects and items. J. Mem. Lang..

[13] Bates D., Maechler M., Bolker B., Walker S. (2015). Fitting linear mixed-effects models using Lme4. J. Stat. Softw..

[14] R Core Team (2019). R: A Language and Environment for Statistical Computing.

[15] Moore B.J., White S., Washington R., Coenen N., Elixhauser A. (2017). Identifying Increased Risk of Readmission and In-hospital Mortality Using Hospital Administrative Data: The AHRQ Elixhauser Comorbidity Index. Med. Care.

[16] Diegoli H., Magalhães P.S.C., Martins S.C.O., Moro C.H.C., França P.H.C., Safanelli J., Nagel V., Venancio V.G., Liberato R.B., Longo A.L. (2020). Decrease in Hospital Admissions for Transient Ischemic Attack, Mild, and Moderate Stroke During the COVID-19 Era. Stroke.

[17] Axenhus M., Schedin-Weiss S., Tjernberg L., Winblad B. (2024). The impact of the COVID-19 pandemic on neurosurgery in the elderly population in Sweden. BMC Public Health.

[18] Szarek D., Miękisiak G., Szmuda T., Fercho J., Pettersson S., Kipiński L. (2023). The impact of the COVID-19 pandemic on the number of brain tumor surgeries in Poland: A national database study. Adv. Clin. Exp. Med..

[19] Tambuyzer T., Vanhauwaert D., Boterberg T., De Vleeschouwer S., Peacock H.M., Bouchat J., Silversmit G., Verdoodt F., De Gendt C., Van Eycken L. (2023). Impact of the COVID-19 Pandemic on Incidence and Observed Survival of Malignant Brain Tumors in Belgium. Cancers.

[20] Ikawa F., Isobe N., Michihata N., Oya S., Ohata K., Saito K., Yoshida K., Fushimi K., Yasunaga H., Tominaga T. (2021). In-Hospital Complications After Surgery in Elderly Patients with Asymptomatic or Minor Symptom Meningioma: A Nationwide Registry Study. World Neurosurg..

